# Automated customized retrieval of radiotherapy data for clinical trials, audit and research

**DOI:** 10.1259/bjr.20170651

**Published:** 2018-01-31

**Authors:** Marina Romanchikova, Karl Harrison, Neil G Burnet, Andrew CF Hoole, Michael PF Sutcliffe, Michael Andrew Parker, Rajesh Jena, Simon James Thomas

**Affiliations:** 1 Department of Medical Physics & Clinical Engineering, Cambridge University Hospitals, Cambridge, UK; 2 Department of Physics, University of Cambridge, Cambridge, UK; 3 Department of Oncology, University of Cambridge, Cambridge, UK; 4 Department of Engineering, University of Cambridge, Cambridge, UK

## Abstract

**Objective:**

To enable fast and customizable automated collection of radiotherapy (RT) data from tomotherapy storage.

**Methods:**

Human-readable data maps (TagMaps) were created to generate DICOM-RT (Digital Imaging and Communications in Medicine standard for Radiation Therapy) data from tomotherapy archives, and provided access to “hidden” information comprising delivery sinograms, positional corrections and adaptive-RT doses.

**Results:**

797 data sets totalling 25,000 scans were batch-exported in 31.5 h. All archived information was restored, including the data not available via commercial software. The exported data were DICOM-compliant and compatible with major commercial tools including RayStation, Pinnacle and ProSoma. The export ran without operator interventions.

**Conclusion:**

The TagMap method for DICOM-RT data modelling produced software that was many times faster than the vendor’s solution, required minimal operator input and delivered high volumes of vendor-identical DICOM data. The approach is applicable to many clinical and research data processing scenarios and can be adapted to recover DICOM-RT data from other proprietary storage types such as Elekta, Pinnacle or ProSoma.

**Advances in knowledge:**

A novel method to translate data from proprietary storage to DICOM-RT is presented. It provides access to the data hidden in electronic archives, offers a working solution to the issues of data migration and vendor lock-in and paves the way for large-scale imaging and radiomics studies.

## Introduction

Collection and harmonization of radiotherapy (RT) data for clinical trials, quality assurance (QA) and research purposes is a non-trivial task that requires integration of numerous software systems and data types.^1–3^ The retrieval pipeline includes unarchiving, restoration on clinical databases, manual selection via graphical user interface and rearchiving. The complexity of the process frequently leads to errors and incomplete restores. Older data may no longer be readable and accumulate in “data graveyards”.

The VoxTox study includes 873 patients treated with helical tomotherapy between 2007 and 2017.^4^ The data exceed 300,000 files archived in several proprietary formats. To collect and harmonize these data with minimal impact on clinical workflow, we developed a framework to translate proprietary-formatted data into the DICOM-RT objects RT-Plan, RT-Structure Set, RT-Image and RT-Dose. We also enabled access to the information “hidden” in patient archives, *e.g.* not available for conventional DICOM export.^5^ The method can be adapted to translate other vendor data formats into DICOM-RT to suit the purposes of different centres.

## Methods and materials

Similar to other vendors such as Pinnacle, ProSoma and Elekta, TomoTherapy © electronic archives comprise structured text and raw data (detector sinograms, planned fluence, images and voxel doses). We developed the data translation software following the analysis, collection, modelling, software implementation and testing cycle stages described below.

### Analysis of vendor storage formats

Tomotherapy patient archives comprise files in the extensible markup language (XML) that hold RT treatment and metadata, and binaries containing the imaging, dose and detector data stored as “<UID>.img/bin/dpe” files.^6, 7^ Data fragments in XML master-files are linked via DICOM unique identifiers (UIDs). Three major versions of storage formats were identified. The only version-related change in raw data formats was observed in the plan sinogram storage versions 3.xx/4.xx. Imaging, detector and dose data were stored unmodified, with the exception of the plan CT images. The latter were reshaped (a) following the insertion of the tomotherapy couch and (b) during the DICOM export.

### Data collection

A database of 20 patients treated between 2008 and 2012 was established to cover all storage formats and transformation scenarios. The database included the electronic vendor archives from two tomotherapy units, the vendor-exported DICOM-RT as well as the positional corrections manually recorded by the treatment radiographers.

### Modelling

To translate vendor storage into DICOM-RT, we created a collection of XML files termed “TagMaps”. Each TagMap listed the DICOM tags for one DICOM-RT type. The TagMaps were created using the dcm2xml utility from the dcm4che2 DICOM toolkit.^8^ Tag contents were set to contain queries to locate the corresponding data in the vendor storage using XPath 2.0 language.^9^ We tested the queries using the XMLTools Notepad ++ plug in.

DICOM sequences such as *ReferencedImageSequence* contain many elements with identical structures. Therefore, it is sufficient to model only one element per sequence, and to use it as a template. The number of the elements to be generated was derived from a dedicated tag. The algorithm is described in Supplementary Material 1 (Supplementary material available online).

Three sets of TagMaps were produced to map the storage versions 3.xx, 4.0 and 4.2+. The TagMaps were based on the vendor’s DICOM Conformance Statement.^10^

### Implementation

**Figure 1.**
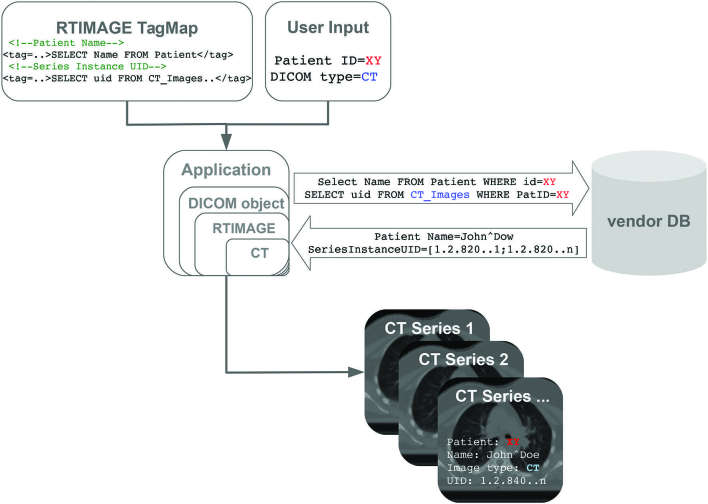
Generation of DICOM-RT objects using TagMaps. The application receives user input to retrieve CT data for patient XY and uses the RTIMAGE TagMap to generate a new DICOM object. At this stage, the DICOM object contains the queries to the vendor storage. The application then executes the queries on the vendor storage and overwrites the DICOM object with the retrieved data. The resulting DICOM objects are saved as DICOM CT image series.

The software to read user inputs, locate the patient data, run XPath queries and write the DICOM-RT objects was implemented in Java 1.7 (Figure 1). The dcm4che2 toolkit was used to generate DICOM objects from TagMaps.

**Figure 2.**
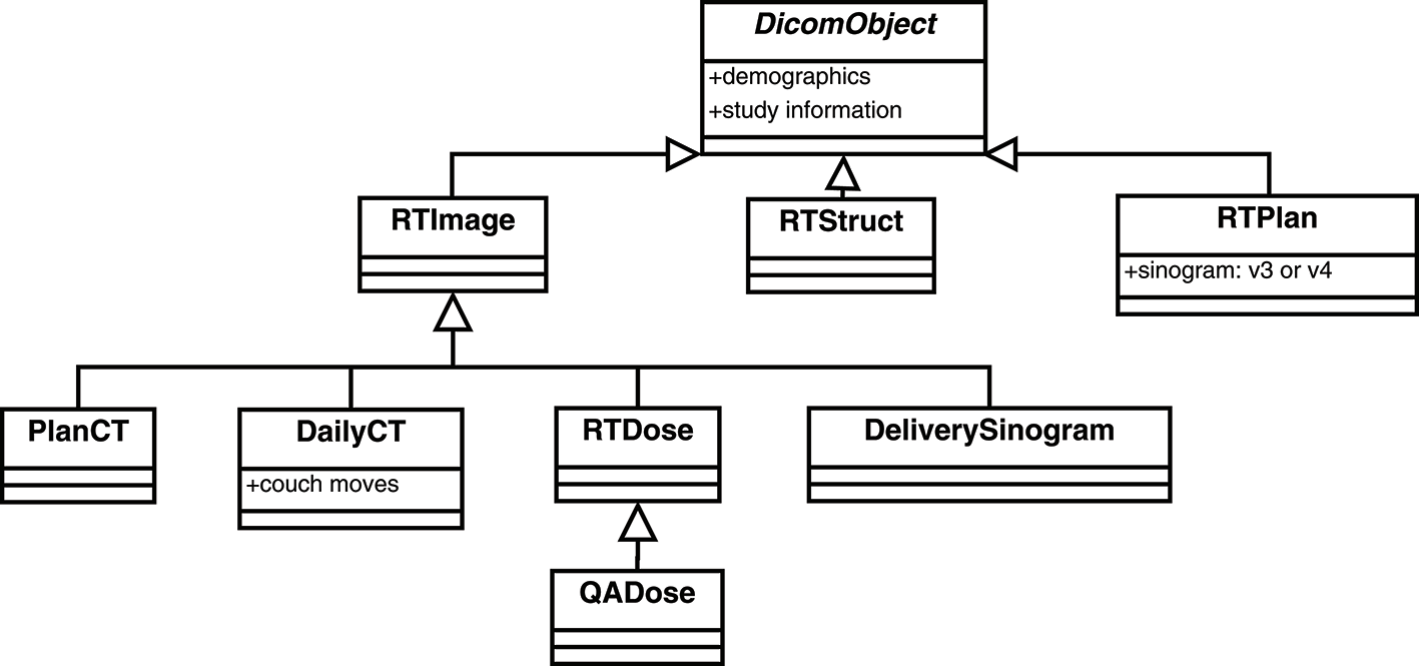
DICOM object hierarchy in the application.

A collection of DICOM classes derived from the parent class *DicomObject* was created to handle object-specific raw data transformations (Figure 2).

The raw data transformations included type conversions, reshaping, splitting and arithmetic manipulations. These transformations can be (a) implemented in the translation software or (b) encoded in TagMaps. We performed simple transformations such as centimetre-to-millimetre conversions in the TagMaps, and offloaded more complex manipulations such as image padding into the corresponding DICOM classes.

#### Plan CT export

During treatment planning, the image of the diagnostic scanner couch on the planning CT is replaced by the tomotherapy couch image. To incorporate the tomotherapy couch image into the planning CT, vertical and horizontal image padding may be necessary. Using the DB defined during the mining stage, we reverse-engineered the padding algorithm and implemented it in the RT-Image class, generic to all RT-Image objects.

####  RT-Dose export

Tomotherapy dose data are stored as voxel doses in gray in 32-bit floating point format. The exported RT-Dose converted dose values to 16-bit integers using scaling factors (SFs) to reduce the file size.

$$D_{16}=\frac{D_{32}}{SF}$$

The SFs can be defined arbitrarily, provided that the precision is preserved between conversions. We defined the SF as the ratio of the maximum voxel dose and the maximum of the 16-bit integer number range.

#### Plan sinogram

Plan sinograms are sequences of multileaf collimator (MLC) opening times for each gantry/couch position [ “Control Point” (CP) or “projection” stored as fractions of MLC-open-time/CP. The sinogram storage underwent significant changes between 3.xx and 4.xx: while 3.xx stored sinogram data as MLC-open-time/CP, v. 4.xx stored open-leaves-only cumulative MLC opening times for each leaf. The data types changed from float in 3.xx to double in 4.xx. To handle 3.xx/4.xx sinogram formats, we implemented a private class PlanSinogram encapsulated in the RTPlan class.

PlanSinogram automatically detects and converts 3.xx and 4.xx storage to the DICOM-RT-Plan format.

#### Export of hidden data

The data described in this section are not included in the vendor-exported DICOM-RT.

### Patient position corrections following image guidance

The corrections in the couch/gantry positions during the image guidance (IG) are essential for image registration, QA and audits. In the tomotherapy storage, the corrections are linked to the corresponding IG scan via a UID in the *fullCorrelationDataArray* fragment (Analysis of vendor storage formats). If the communication between the treatment unit and the database is disrupted, the corrections are recorded, but the linking is lost. To relink the data, we defined a fallback procedure that located the IG scan using timestamps. The exported corrections were included into the IG-megavoltage CT (MVCT) scans as private tags.

### Adaptive radiotherapy dose

PlannedAdaptive © software allows dose recalculation on daily IG-MVCT scans to assess dose differences arising from changes in patient’s anatomy. However, the recalculated doses are available only for visual inspection on tomotherapy workstations. To access the recalculated voxel doses, we defined a QADose type based on DICOM RT-Dose. The QADose data were used to validate the dose calculation software.

### Delivery sinogram

During a treatment delivery, the tomotherapy imaging detector is recording a signal that is stored as an average value per projection for each detector channel. These “delivery sinograms” provide valuable information for *in vivo* dosimetry and QA.^11^ Although, from 2014 commercial software exists to analyse the delivery sinograms, it suffers from the same limitations as other graphical user interface-based tools and is unsuitable for automated data analysis.^12^ An RTIMAGE-based TagMap was created to restore the delivery sinograms. The exported sinograms have been used in the department’s QA procedures. Methods for in-treatment dose monitoring using comparison of planned and delivered sinogram data are under development.

### Testing

The exported DICOM data were compared with their vendor-exported counterparts and imported into several commercial and freeware tools. The exported position corrections were compared with the treatment radiographers manual records. For the performance testing, RT-Plan, RT-Dose, CT and MVCT data for five patients were exported using (a) our software and (b) PlanningStation ©. Our software was run on a Dell Precision with 3.3 GHz Intel Xeon CPU and 8 GB RAM. Identical network locations for the input and output data were used for both export methods.

## Results

### DICOM-RT export

**Figure 3.**
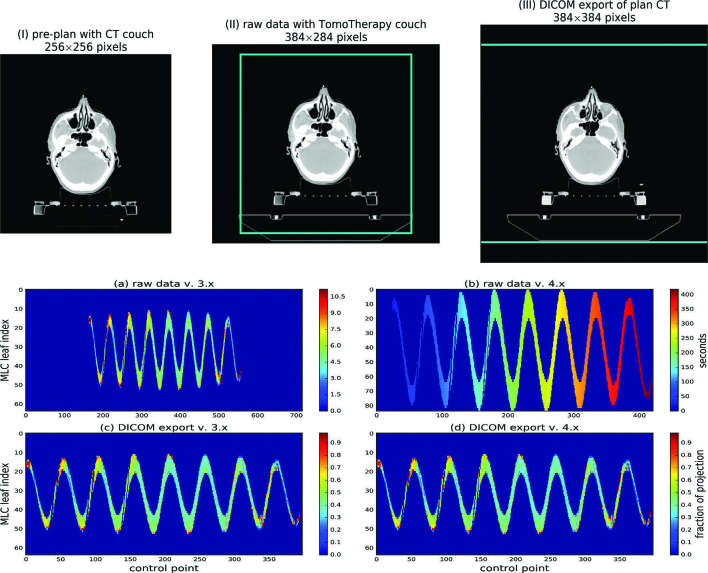
Transformations of binary data for the DICOM export. (I –III) Insertion of the treatment machine couch image into the plan CT. (I) 256 × 256 CT image with CT scanner couch; (II) treatment couch is inserted, the image is enhanced to 384 × 284 pixels to incorporate a larger couch image with preservation of the treatment isocentre; (III) for DICOM export, the image is padded to 384 × 384 pixels. Cyan lines in (II) and (III) mark the image area inherited from the previous processing step. (a –d) Sinogram export in vv 3.xx (a, c) and 4.xx (b, d). (a, b): raw data in vendor storage. (b, d) RTPLAN Tomo Projection Sinogram Data tag (300 D, 10A7). Raw data for v.4.x (b) show leaf opening and closing times for the MLC leaves open during the treatment (MLCs 11 to 52); the sinogram height is number of open leaves × 2. The entries for leaves 1 –10 and 53 –64, that are closed for the entire treatment duration, are not recorded. The left hand-side of (b) depicts 10 s beam warm-up time.

Figure 3 I –III demonstrates transformations of a plan CT image following the couch replacement. We reproduced the changes in the image dimensions for all modification scenarios (no change, vertical/horizontal/vertical & horizontal enhancements). The data exported using our algorithm were identical to the vendor exports. The RT-Plan objects were correctly restored for both underlying binary formats (Figure 3a –d). The TagMap- and vendor-exported RT-Doses showed differences of ± 0.001% of the maximum dose. These were expected from the number rounding during the format conversions (RT-Dose export).

### Performance

The mean export time/data set using PlanningStation was 15 ± 7 min per treatment unit, with 10 min preparation and 5 min of “pure” DICOM export time. Our framework required 30 ± 5 s for DICOM export, and 1 –2 min for the automated data lookup, generation of input files and output QA. The retrieval was automated and ran without operator supervision. The data exported using our software included IG positional corrections (Export of hidden data) that were used to recalculate the delivered radiation doses.

### Automated data retrieval

We extracted 797 patient data sets including over 25,000 daily scans. The retrieval took 31.5 h and ran as a background task concurrent with the routine workload. All archived data were successfully recovered. 88% of the restored data sets were complete; 12% needed additional processing owing to missing archives. The average time to process one data set including creation of text input files, DICOM-RT generation, export, network transfer from the archived storage and testing was 2 ± 0.5 min per patient.

### DICOM conformance

Apart from the insignificant dose differences (DICOM-RT export) and private tag enhancements (Export of hidden data), the DICOM-RT data sets exported using our software were identical to the tomotherapy exports, and preserved the UID links between the files. The generated DICOM-RT were tested and accepted in the clinical and research software packages summarized in Table 1.

**Table 1.** Compatibility of extracted DICOM data with commercial and open-source software

****	**CT**	**MVCT**	**RT-Plan**	**RT-Dose**	**RT-Struct**
ProSoma 3.3	+^*a*^	+^*a*^	n/a	+^*a*^	+^*a*^
Pinnacle 9.8	+	+	n/a	+	+
OnQ RTS	+^*b*^	+^*b*^	n/a	+*^b^*	+*^b^*
RayStation 3.5	+	+	n/a	n/a	+
Mirada Medical XD3	+	+	n/a	n/a	+
DCMTK 3.6.0 –15	+	+	+	+	+
Dicompyler	+	+	+	+	+
ImageJ	+	+	n/a	+	n/a
CheckTomo	+	+	+	+	+

^*a*^ProSoma software removed the StudyTime DICOM tag during the import.

^*b*^Image data was accepted in Explicit LittleEndian transfer syntax only.

## Discussion

Compared with the vendor’s DICOM-RT export, our software was more than 10 times faster. We attribute this to the use of state-of-the-art software libraries and bypassing the internal conversions in the vendor software. The exported DICOM-RT objects were correctly linked and compatible with a number of RT software tools. We found that some software packages may alter the contents of DICOM objects during import/export, and recommend thorough testing for each DICOM-RT type and software package to avoid data losses or corruptions. The standardized and customizable nature of the extracted DICOM-RT data make the mapping software a useful tool in clinical RT trials.

However, validation must be performed to ensure that the extracted data are identical to those extracted using trial-approved methods. Examples of such validation are given in the section "DICOM conformance" and Table 1. While this work was focussed on the translation of tomotherapy storage, the method can be generalized to handle other storage types including relational databases (Supplementary Material 1). MOSAIQ oncology information system (OIS) stores data in a Structured Query Language (SQL) database. Example 1 presents a TagMap that locates the RT-Treatment Record data in the MOSAIQ-DB using SQL queries.

The evaluation is performed in stages: first, all studies associated with a patient are found; then, all study series are located; then, the RTPLAN object linked to the series is located.

### Example 1

RT-Treatment Record TagMap for MOSAIQ 2.64. Table and column names are altered.

<dicom>

<!--Patients Name-->

<attr tag= “00100010” > SELECT FIRST_NAME, LAST_NAME FROM Patient WHERE Patient.ID = ? </attr>

<!--Study Instance UID-->

<attr tag= “0020000D ”>SELECT s.StudyInstanceUID FROM DCMStudy S INNER JOIN Patient P ON P.Pat_ID1 AND P.ID = ? </attr>

<!--Series Instance UID-->

<attr tag= “0020000E ” vr= “UI ”>SELECT SeriesInstanceUID FROM DCMSeries D WHERE D.DCMStudy_ID = ? </attr>

<!--Referenced RT Plan Sequence-->

<attr tag= “300C0002 ” vr= “SQ ”>

<item>

<!--Referenced SOP Class UID (constant value)-->

<attr tag= “00081150 ” vr= “UI ”>1.2.840.10008.5.1.4.1.1.481.5</attr>

<!--Referenced SOP Instance UID-->

<attr tag= “00081155 ” vr= “UI ”>SELECT PlanInstanceUID FROM TreatmentList T WHERE T.ID = ? AND T.ImageSeriesUID = ? </attr>

</item>

</attr>

</dicom>

Following the process described in Methods and materials, the application would generate a blank DICOM file from the TagMap, establish the connection to MOSAIQ database and populate the DICOM object by evaluating the SQL queries.

In our experience, the software development and data mapping require 3 –12 months, depending on the existing documentation and expertize, while updates and addition of new data types take from a few hours to a few days. We consider this time investment worthwhile. The mapping software can retrieve incomplete or historic data sets, provides enriched information about the RT treatment (Export of hidden data) and future-proofs the patient data by converting it from proprietary formats into the DICOM-RT. The latter is of particular value for radiomics applications, as these require large volumes of standardized imaging data to build reliable models.^13^

## Conclusions

The TagMap framework to translate tomotherapy electronic archives to customized DICOM-RT objects is described. The framework enabled access to the “hidden ” data including IG positional corrections, delivery sonograms and “adaptive-RT ” doses. We restored historic data incompatible with the current vendor software. To our knowledge, this is the first implementation of large-scale automated retrieval of tomotherapy data. TagMaps provide human-readable data models that can be changed on-the-go to alter the data selection criteria or to add/remove contents to the exported DICOM-RT data. Such flexibility is impossible to achieve in the commercial environment, owing to long feedback loops between users and developers, and to the complexity of software development under the Medical Device Directive.^14^

The method can be used as a customizable alternative to vendor software for DICOM-RT export, as well as a standalone tool to collect the data disseminated across multiple locations and stored in various formats.
